# Fingerprints of magnetoinduced charge density waves in monolayer graphene beyond half filling

**DOI:** 10.1038/s41598-022-26122-0

**Published:** 2022-12-15

**Authors:** Felix Hoffmann, Martin Siebert, Antonia Duft, Vojislav Krstić

**Affiliations:** Department of Physics, Friedrich-Alexander-University (FAU) Erlangen-Nürnberg, Staudtstraße 7, 91058 Erlangen, Germany

**Keywords:** Condensed-matter physics, Electronics, photonics and device physics, Condensed-matter physics

## Abstract

A charge density wave is a condensate of fermions, whose charge density shows a long-range periodic modulation. Such charge density wave can be principally described as a macroscopic quantum state and is known to occur by various formation mechanisms. These are the lattice deforming Peierls transition, the directional, fermionic wave vector orientation prone Fermi surface nesting or the generic charge ordering, which in contrast is associated solely with the undirected effective Coulomb interaction between fermions. In two-dimensional Dirac/Weyl-like systems, the existence of charge density waves is only theoretically predicted within the ultralow energy regime at half filling. Taking graphene as host of two-dimensional fermions described by a Dirac/Weyl Hamiltonian, we tuned indirectly the effective mutual Coulomb interaction between fermions through adsorption of tetracyanoquinodimethane on top in the low coverage limit. We thereby achieved the development of a novel, low-dimensional dissipative charge density wave of Weyl-like fermions, even beyond half filling with additional magneto-induced localization and quantization. This charge density wave appears both, in the electron and the hole spectrum.

## Introduction

A charge density wave (CDW) is a collective state of interacting fermions^1–3^ principally describable as a quantum state with a macroscopic phase^2,4–6^. Such condensate is characterized by a periodic modulation of the charge carrier density showing dissipative electronic transport signatures^3,7–10^. Two frequent CDW formation mechanisms are the Peierls distortion^11^, and Fermi surface nesting^11,12^. Another option is generic charge ordering due to the effective Coulomb interaction between the fermions^11^. For all these reasons CDWs offer various potential applications in quantum memory components^13,14^ and quantum computing devices^15,16^. Consequently, the theoretical description as well as experimental study of CDW state formation in low-dimensional (low-D) or unconventional fermionic systems, composed of for instance, 3D Weyl or low-D Dirac and Weyl-like fermions, continues to attract significant attention^17–27^. 2D examples for the latter are found in Kagomé, Lieb and hexagonal lattices^28,29^. Kagomé lattices are generally only realized in layered structures and despite a well established theoretical framework^30,31^ and a set of experimental reports^32–34^ about CDW formation, a number of open questions remains unresolved. Similarly, electronic Lieb lattices and CDW formation therein have been discussed from a theoretical viewpoint^35,36^, yet their experimental realization has proven to be highly challenging^37–39^. Finally, in the intriguing case of 2D hexagonal lattices, only theoretical works have been conducted^20–23^. These works revealed that CDWs could form out of the semimetallic phase by generic charge ordering provided the corresponding on-site and long-range repulsions could be tuned accordingly relative to the kinetic energy^20–23^. However, this is only predicted in the ultralow energy limit at half filling, that is, at the charge neutrality point (CNP). Notably, the formation of such charge ordered state could theoretically also be achieved beyond half filling under the provision of additional magneto-induced localization. That is, as long as the ratio of magnetic length $l_{B}$ and the mean distance between charge carriers $r_{s}$ is smaller or equal to unity^40^. The development of a CDW in 2D Weyl-like fermionic systems in a magnetic field thus depends in general on the effective pairwise Coulomb interaction relative to the kinetic energy, $r_{s}$ and $l_{B}$^41^. Here we demonstrate the formation of an unprecedented CDW of the 2D Weyl-like fermions hosted in graphene beyond half filling in the high magnetic field limit. Remarkably, this state appears within the electron as well as the hole spectrum. Due to the dissipative nature of electronic transport associated with CDWs^3,7^, the magnetotransport characteristic of this CDW is a peaking longitudinal resistivity accompanied by an unconventionally valued transverse conductivity plateau quantized in $\frac{{e^{2} }}{h}$ (e: elementary electric charge, *h*: Planck constant). We monitored the evolution of this signature in samples of different physisorbed tetracyanoquinodimethane (TCNQ) load and, in part, varying magnetic field.

Graphene, by default a semimetal^42,43^, provides through its bipartite hexagonal lattice structure a perfect model system for the exploration of low-D charge ordering phenomena. In the low-energy regime, it can be described by a massless 2D Dirac/Weyl Hamiltonian of the form^44^

1 $$H = \hbar \nu_{F} \;{{\varvec{\upsigma}}} \cdot {\varvec{k}} = \hbar \nu_{F} \left( {\begin{array}{*{20}c} {0} & {k_{x}-ik_{y}} \\ {k_{x}+ik_{y}} & {0} \\ \end{array} } \right)$$

with $\hbar$ the reduced Planck constant, $\nu_{F}$ the Fermi velocity, ${{\varvec{\upsigma}}} = \left( {\sigma_{x}} ,\user2{ }\sigma_{y} \right)$ the Pauli matrices vector and ${\varvec{k}}$ the wavevector^44^. That is, both sublattices contribute to the electronic states near the points in reciprocal space where valence and conduction band touch ($K$ and $K^{\prime }$ points, respectively). The sublattices’ contributions are reflected in the circumstance, that the electric wavefunctions are two-component spinors^45^. These wavefunctions near $K$ and $K^{\prime }$ are given by $$\psi_{{K,K^{\prime } }} \left( {\varvec{k}} \right) = \frac{1}{\sqrt 2 } \left( {\begin{array}{*{20}c} 1 \\ {se^{{ \pm i\theta_{\varvec{k}} }} } \\ \end{array} } \right)$$, where the $s = \pm 1$ is the band index, the $\pm$ in the exponent distinguishes $K$ and $K^{\prime }$ and $\theta_{\varvec{k}}$ is the polar angle of ${\varvec{k}}$^43^. Also, Eq. (1) shows, that the dispersion relation $E\left( {\varvec{k}} \right) = \hbar v_{F} \left| {\varvec{k}} \right|$ is linear in this limit.

**Figure 1:**
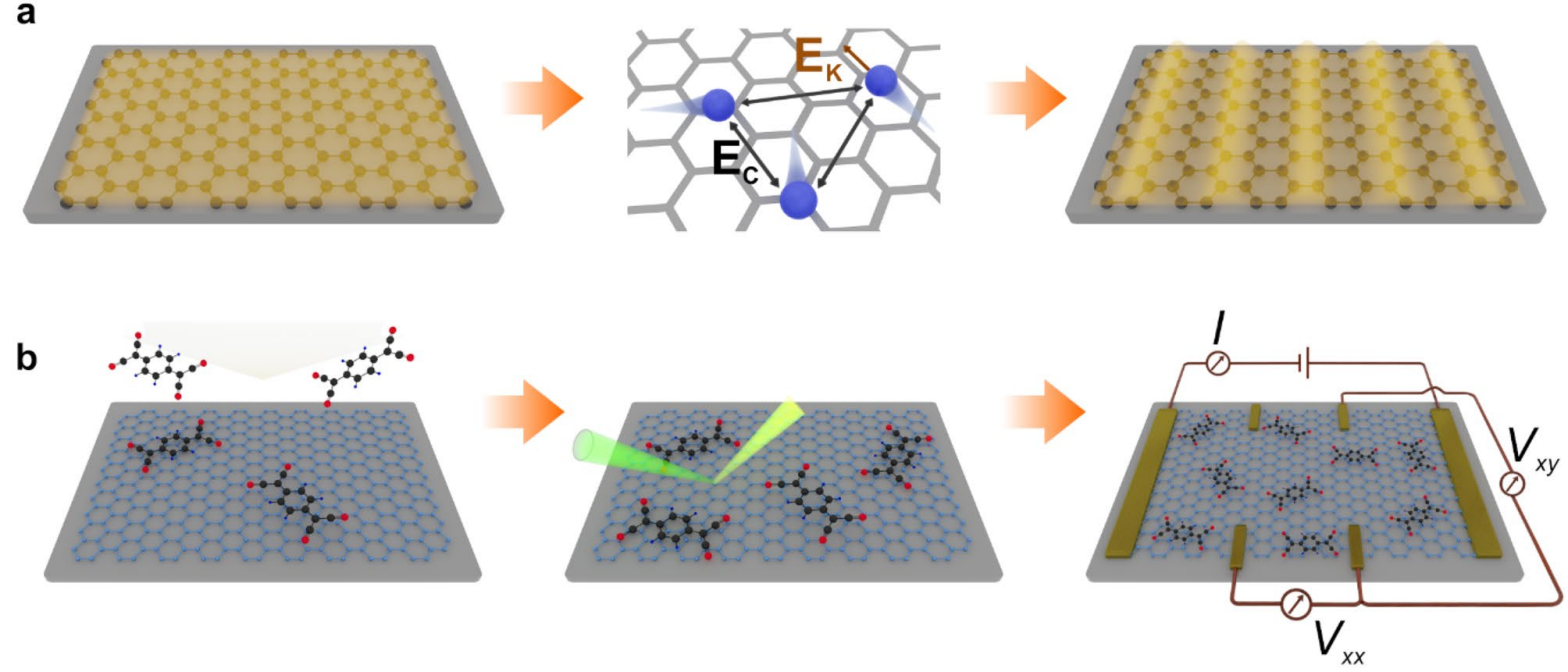
Schematic illustration of a generic charge ordered CDW formation & principle sample processing: (**a**) Schematic (not to scale) of a CDW formation mechanism due to generic charge ordering. Left: graphene (black lattice) with an unmodulated electron density (yellow). Middle: the magnitude of the effective mutual Coulomb interaction between charge carriers (blue spheres) relates to the ratio of their pairwise Coulomb repulsion $E_{C}$ (black arrows) and their kinetic energy $E_{K}$ (dark orange arrow) (cf. Supplementary Note 1)^46^. Right: Once $E_{C}$ prevails over $E_{K}$, a CDW state exhibiting a periodic modulation of the charge density can principally develop. Here, for instance and for pure visualization purposes, an arbitrarily chosen one-dimensional CDW case is schematically illustrated^9,10^. (**b**) Principle workflow (not to scale) of the experimental approach. Left: Random deposition of TCNQ molecules (black: carbon, red: nitrogen, blue: hydrogen) on the graphene lattice (light blue) from the gas phase. Middle: Characterization of the samples via micro-Raman spectroscopy (incident and scattered light indicated by cones). Right: A sample electrically contacted in Hall-bar geometry and principle setup for transport measurements. For magnetotransport measurements, the magnetic field is oriented perpendicular to the sample plane.

However, due to this linear dispersion relation the effective Coulomb-interaction, described by the ratio $\frac{{E_{C} }}{{E_{K} }}$ of the pairwise Coulomb repulsion $E_{C}$ and the particles kinetic energy $E_{K}$, is solely dependent on the dielectric function ϵ of the system . Thus, it cannot be tuned via charge-carrier density increase (cf. Supplementary Note 1)^46^. That is, techniques to adjust the carrier density like gating or the substitution or intercalation of dopants are genuinely not viable to induce CDW formation in such systems. Nevertheless, we achieved the CDW formation in graphene by physisorbing TCNQ on top^47–49^ in the low coverage limit: The presence of the TCNQ molecules modulates the screening properties and therefore ϵ of the system. Following this strategy, we tuned ϵ such that the favorable range for CDW formation ($\frac{{E_{C} }}{{E_{K} }} \gtrsim$ 1; cf. Supplementary Note 1) is reached (Fig. 1).

## Results

### Raman characterization of graphene with deposited TCNQ

**Figure 2:**
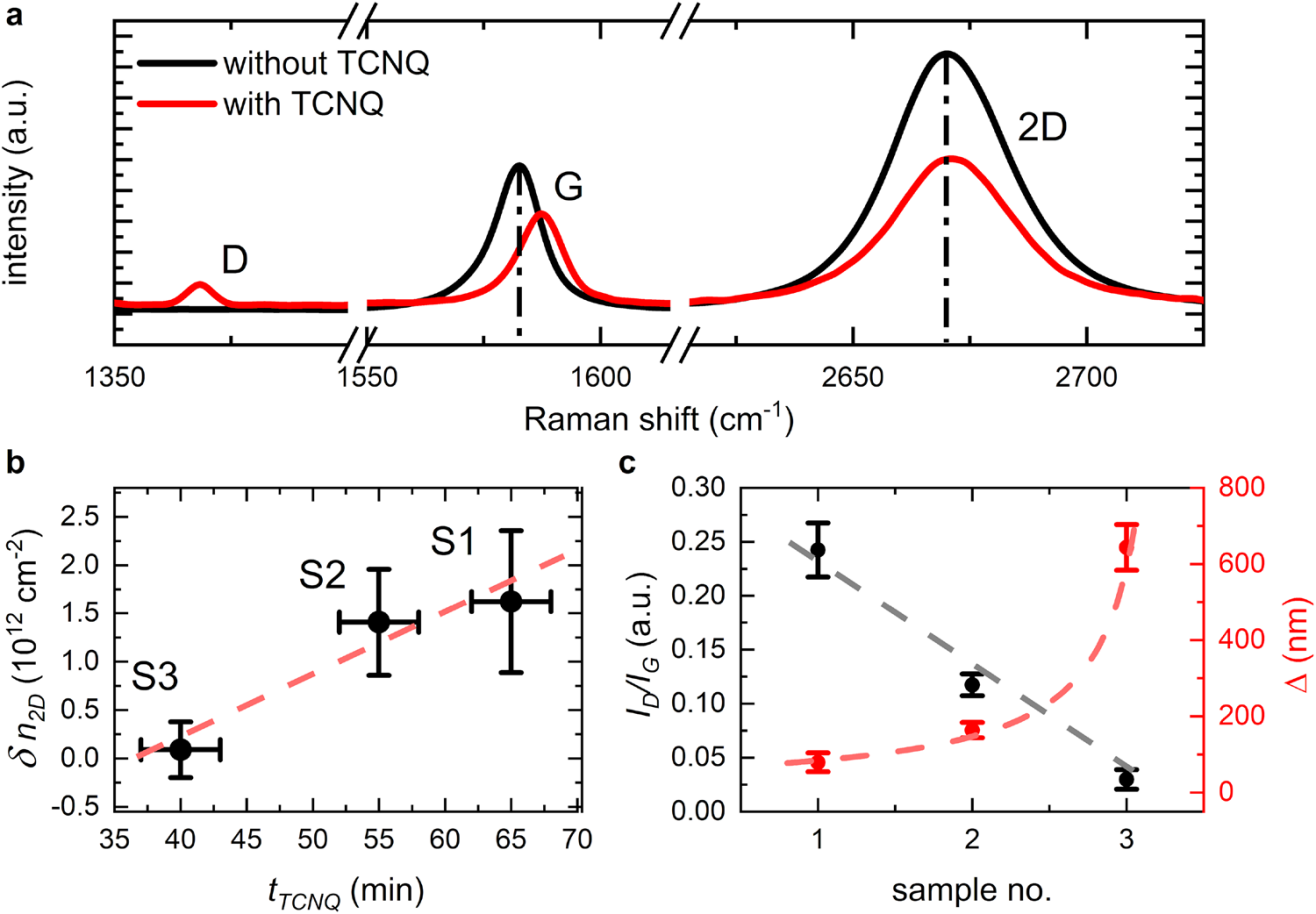
Micro-Raman characterization of graphene/TCNQ samples: (**a**) Exemplary Raman spectrum of a graphene sample, before and after TCNQ deposition. Displayed spectra are averages of experimental Raman maps (> 20 individual spectra). With TCNQ a clear increase in D-band intensity is observed. The shift of G-band ($+ 4\;{\text{cm}}^{ - 1} \pm 1\;{\text{cm}}^{ - 1}$) towards higher wavenumbers indicates the electron transfer to the TCNQ^51^. Regarding the 2D band peak position, only a small shift seems to be apparent. (**b**) Change in charge carrier density $\delta n_{2D}$ estimated from the G-band shift as a function of TCNQ evaporation time $t_{TCNQ}$. Error bars are resulting from uncertainties in the fit procedure and random temperature fluctuations in heating and cooling phases (cf. Method section). The red dashed line serves as a guide to the eye. (**c**) Ratio of D- and G-band intensity $\frac{{I_{D} }}{{I_{G} }}$ for the three samples (left axis). The larger $\frac{{I_{D} }}{{I_{G} }}$, the smaller the average spacings within the random TCNQ distribution as reflected by $\Delta$ (right axis)^54^. Dashed line serves as guide to the eye.

For our case of 2D Weyl-like fermions hosted in graphene, randomly deposited dopant molecules are excellently suited to modulate the screening within the system. In particular TCNQ offers a high electron affinity with a charge transfer of ~ 0.3 electron per molecule^47–49^. The charged molecule induces a perturbation within the electrostatic potential landscape provided by the graphene lattice, modulating the dielectric function ϵ of the system^46,50^. In addition, the TCNQ-graphene distance is found to be ~ 3 Å^47–49^, which together with TCNQ’s alike dimensions and symmetry to the hexagonal graphene lattice, implies negligible lattice deformations^47–49^ preserving the 2D Weyl-like nature of the fermions. We note, that it can be envisioned, that in principle any molecular structure with a sufficiently high charge transfer and no detrimental impact on the graphene lattice structure is suitable, too (e.g. tetrathiafulvalene). Following this line, we prepared three TCNQ/graphene samples (S1 to S3) with different amounts of TCNQ deposited (variation of the evaporation time $t_{TCNQ}$ from 65, 55 to 40 min, respectively). These we characterized using micro-Raman spectroscopy before and after TCNQ deposition to obtain a measure of the overall charge-transfer (doping) and the average spacings within the random TCNQ distribution. Figure 2a shows the spectra of an exemplary graphene flake. After TCNQ deposition, a shift of the G-band peak position towards greater wavenumbers was found which is characteristic for p-type doping^51^. The shift in the 2D-band peak position is less pronounced, however, it also indicates, that the overall lattice structure of the graphene is not strongly compromised by the TCNQ, as expected^52,53^. From the G-band shift, the change in charge carrier density $\delta n_{2D}$ can be estimated (*cf*. Methods section). In Fig. 2b $\delta n_{2D}$ is plotted against $t_{TCNQ}$. As expected, $\delta n_{2D}$ increases with the amount of TCNQ deposited. Also, the D-band intensity $I_{D}$ augments in all samples, indicative of more points of broken translation invariance. The average spacing of such points $\Delta$ can be estimated by ${ }\left[ {2.4 \times 10^{ - 10} \;{\text{nm}}^{ - 3} } \right] \cdot\uplambda ^{4} \cdot \left( {\frac{{I_{D} }}{{I_{G} }}} \right)^{ - 1}$ (Fig. 2c)^54^. Here $I_{G}$ is the G-band intensity and $\lambda$ = 532 nm the excitation wavelength. Importantly, the observed $\delta n_{2D}$ and the found $\Delta$ in the range from less than hundred to several hundred nm are consistent with a low TCNQ coverage of less than a monolayer. This corroborates that the 2D Weyl-like fermionic nature is overall preserved, also considering the length of the graphene unit cell vector^55^ of 2.46 Å. The sample mobilities (which we will turn to next) corroborate this conclusion.

### Electrical response of graphene/TCNQ samples with different TCNQ load

**Figure 3:**
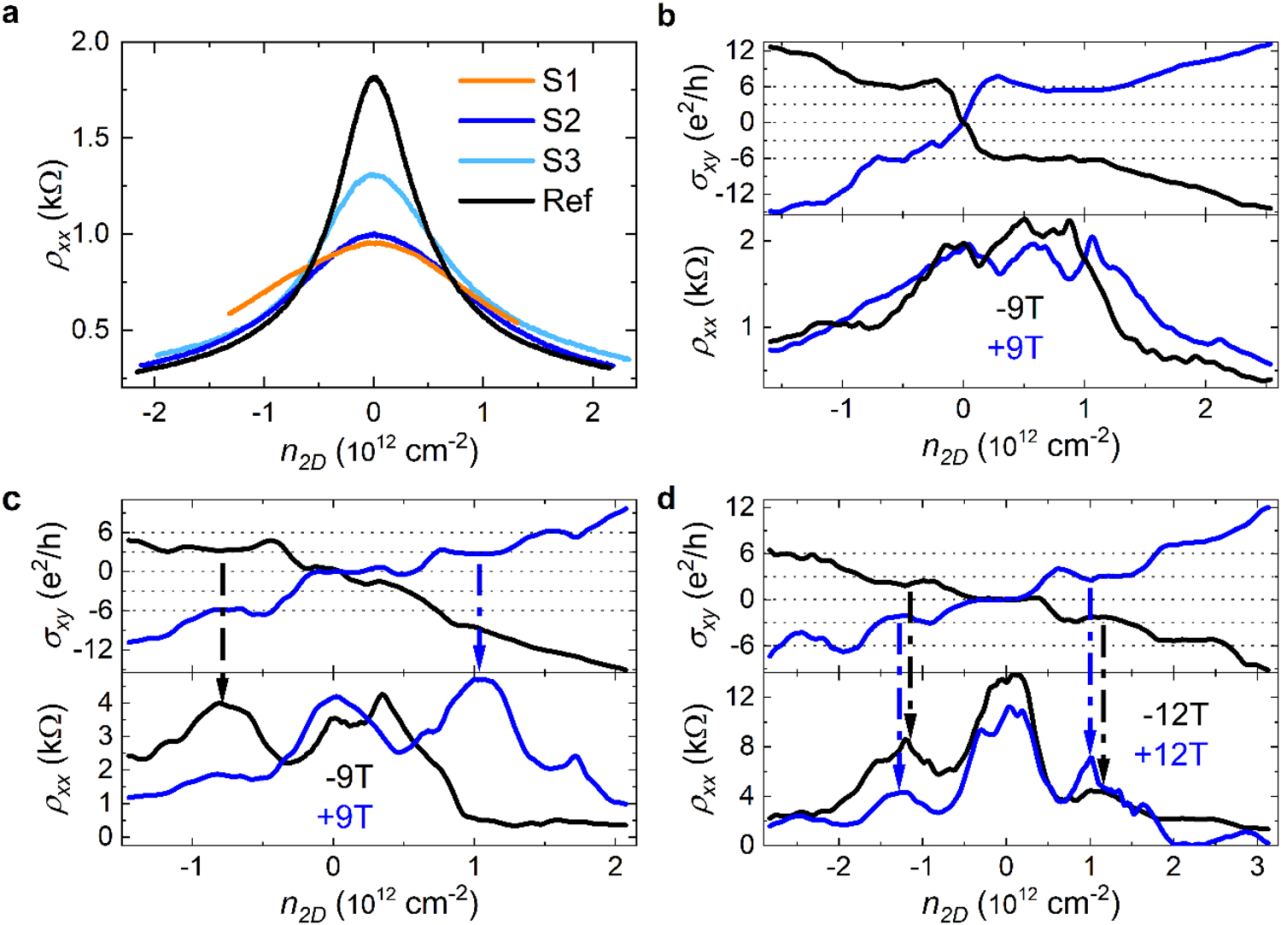
Electrical (magneto-)transport measurements of graphene/TCNQ samples at room temperature and liquid helium temperature: (**a**) Longitudinal resistivity $\rho_{xx}$ at room temperature of all samples including a reference sample with no TCNQ deposited. The ambipolar behavior of graphene is clearly found in each. Magnetotransport measurements (**b**–**d**) of the samples S1 to S3 were carried out at liquid helium temperature. Transverse conductivity $\sigma_{xy}$ and $\rho_{xx}$ measured at magnetic fields of $\pm$ 9 T (S1 & S2) and $\pm$ 12 T (S3). (**b**) S1 shows plateau indicating kinks with zero $\sigma_{xy}$ and a corresponding maximal $\rho_{xx}$ at the CNP. We note, that for a considerably disordered system as S1, an asymmetric transport behavior with respect to the CNP showing additional features can be expected^56^. (**c**) S2 exhibits a clear local maximum in $\rho_{xx}$ accompanied by a pronounced plateau at $\sigma_{xy}$ = $0\frac{{e^{2} }}{h}$ for both field directions. Additionally, maxima in $\rho_{xx}$ backed by unconventional plateaus at $\sigma_{xy} = + 3\,\frac{{e^{2} }}{h}$ are observed (indicated by dashed arrows). Finally, plateaus at $\sigma_{xy} = \pm 6\frac{{e^{2} }}{h}$ appear together with local minima in $\rho_{xx}$ for large $n_{2D}$, designating them as quantum Hall states. (**d**) For S3, plateaus with $\sigma_{xy}$ = $0\frac{{e^{2} }}{h}$ and maximal $\rho_{xx}$ are present at the CNP for both field directions. Additional unconventionally valued plateaus appear at $\sigma_{xy} = + 3\,\frac{{e^{2} }}{h}$ and $\sigma_{xy} = - 3\,\frac{{e^{2} }}{h}$, together with maxima in $\rho_{xx}$ (dashed arrows) also for both field directions. Furthermore, all plateaus with $\sigma_{xy} = \pm 6\frac{{e^{2} }}{h}$ are accompanied by local minima in $\rho_{xx}$, identifying them as quantum Hall states.

The electrical measurements were carried out at room temperature and liquid helium temperature at magnetic field strengths $\left| {\vec{B}} \right| = B =$ 9 and 12 T (S1, S2 and S3, respectively) in Hall configuration. In the following we denote by + *B* and –*B* the relative orientation of the field to the sample plane (out of and into the sample plane, respectively). In Fig. 3a, the longitudinal resistivity $\rho_{xx}$ at room temperature of all samples and a reference sample without TCNQ are shown. In all samples the characteristic ambipolar field-effect in $\rho_{xx}$ with a clearly defined CNP is found reflecting the 2D Weyl-like nature of the fermions^44,55^. The impact of the TCNQ deposition can be traced by the charge carrier mobility $\mu_{e/h}$ (*e* electrons; *h* holes)^56,57^. Starting from the reference sample, $\mu_{e/h}$ decreases as expected monotonically with increasing amount of TCNQ deposited (cf. Raman results). The specific values are: $\mu_{e/h} = \left( {4040 \pm 83} \right) / \left( {1950 \pm 44} \right)\frac{{{\text{cm}}^{2} }}{{{\text{Vs}}}}$ (S1), $\mu_{e/h} = \left( {3880 \pm 78} \right) / \left( {4270 \pm 92} \right)\frac{{{\text{cm}}^{2} }}{{{\text{Vs}}}}$ (S2), $\mu_{e/h} = \left( {4740 \pm 142} \right) / \left( {4900 \pm 228} \right)\frac{{{\text{cm}}^{2} }}{{{\text{Vs}}}}$ (S3) and $\mu_{e/h} = \left( {9730 \pm 63} \right) / \left( {10600 \pm 65} \right)\frac{{{\text{cm}}^{2} }}{{{\text{Vs}}}}$ (reference). Likewise, the $\rho_{xx}$ maximum lessens as expected due to the TCNQ load^55^. We now turn to the magnetotransport at liquid helium temperature for these samples. First, we address S1, which has the highest amount of TCNQ deposited ($\Delta \approx$ 80 nm). In Fig. 3b, $\sigma_{xy}$ and the corresponding $\rho_{xx}$ are shown as a function of $n_{2D}$ for magnetic fields *B* = $\pm$ 9 T. A kink at the CNP with zero $\sigma_{xy}$ is present for both magnetic field directions, indicating the potential development of a plateau. This falls within a maximal region of $\rho_{xx}$, which would be the signature of a CDW state. However, charged impurities distort and can impede the formation of collective modes like CDWs significantly^3,58^. More explicitly, there is a system specific critical ionic impurity concentration $n_{i}^{\left( c \right)}$, beyond which no CDW can exist or develop (addressed further in the “Discussion” section)^58^.

Having the maximal $\rho_{xx}$ at the CNP, accompanied by a kink at $\sigma_{xy}$ = 0 $\frac{{e^{2} }}{h}$, we address in the next step sample S2 with a smaller TCNQ density ($\Delta \approx 160\,{\text{nm}}$). This should be less impeding the development of a CDW state as the TCNQ related local charges are further apart. In Fig. 3c again $\sigma_{xy}$ and the corresponding $\rho_{xx}$ are shown. We find for both *B* directions clear local maxima in $\rho_{xx}$ at the CNP. They are accompanied by pronounced plateaus $\sigma_{xy}$ = $0\frac{{e^{2} }}{h}$, respectively, although for negative field somewhat less well developed. Moreover, additional maxima in $\rho_{xx}$ appear together with unconventional plateaus at $\sigma_{xy} = + 3\,\frac{{e^{2} }}{h}$ for both *B* directions, that is, the expected signature of a CDW phase. Additionally, plateaus at $\sigma_{xy} = \pm 6\frac{{e^{2} }}{h}$ appear together with signatures of extended local minima in $\rho_{xx}$ for positive *B*, identifying these plateaus to belong to the quantum Hall phase. Having twice the main characteristics of long-range charge-ordered phases at hand, equally the plateau dependencies on $n_{2D}$ demonstrates the magnetoinduced CDW formation in our system. The transition $\sigma_{xy}$ = 0 to $+ 3\,\frac{{e^{2} }}{h}$ coincides with the complete filling of the n = 0 Landau level (LL) ($n_{2D} \approx \frac{2eB}{h}$). That is, the zero-conductivity plateaus extend over the entire zeroth LL. The transition from $\sigma_{xy}$ = $3\,$ to $6\,\frac{{e^{2} }}{h}$ is found at the complete filling of the n = 0 and n = 1 LL ($n_{2D} \approx \frac{6eB}{h}$) analogous to the 0 to $+ 3\,\frac{{e^{2} }}{h}$ transition. This circumstance together with the sequence of plateaus $0$, $3$ and $6\,\frac{{e^{2} }}{h}$ with increasing $n_{2D}$ disqualifies other conceivable mechanisms such as the quantum Hall insulator or magnetic catalysis (cf. Supplementary Note 3). The Zeeman effect is also excluded as will be shown further below, which finally concludes the formation of two magnetoinduced CDW phases within S2. To further second the CDW formation in our graphene/TCNQ system, a further increase of the TCNQ separation and a reduction of potentially magneto-related detrimental mechanisms is to be followed. This is achieved in S3, where $\Delta$ is of several hundred nm, and in addition increasing the magnetic field strength. In particular, through the latter we reduce the zero-point fluctuations, since a CDW collapses once these become comparable to the CDW periodicity^59^. Specifically, these fluctuations are in a magnetic field limited to the magnetic length $l_{B} = \sqrt {\frac{\hbar }{eB}}$^44^. In other words, clearer and/or more CDW signatures are expected in S3. In Fig. 3d the corresponding $\rho_{xx}$ and $\sigma_{xy}$ are shown, and as anticipated, for *both* field directions, a pronounced maximum in $\rho_{xx}$ is found at the CNP, accompanied by a well developed $\sigma_{xy} = 0\frac{{e^{2} }}{h}$ plateau. Moreover, plateaus at $\sigma_{xy} = + 3\frac{{e^{2} }}{h}$ *and* $- 3\frac{{e^{2} }}{h}$ are identifiable co-existing with strong local maxima in $\rho_{xx}$. This symmetry of plateaus for both, electrons and holes, is crucial. It shows that the experimental observations are not related to the mere energetic position of the TCNQ induced localized states, since these are only located within the hole spectrum about 250 meV deep^47^. It remains to mention that also plateaus with $\sigma_{xy} = \pm 6\frac{{e^{2} }}{h}$ can be identified for both field directions. All these go along with (differently) well developed local minima in $\rho_{xx}$, even getting to $\rho_{xx}$ = 0 for $\sigma_{xy} = + 6\frac{{e^{2} }}{h}$ for positive field. Importantly, despite the differences in S2 and S3, the transition points between the different plateaus occur under the same conditions, that is, appear once the n = 0 LL (0 $\to$ $\pm 3\frac{{e^{2} }}{h}$) is and, both, the n = 0 and n = 1 LLs ($\pm 3$ $\to$ $\pm 6\frac{{e^{2} }}{h}$) are completely filled.

### Magnetic field dependence of plateau transition points

**Figure 4:**
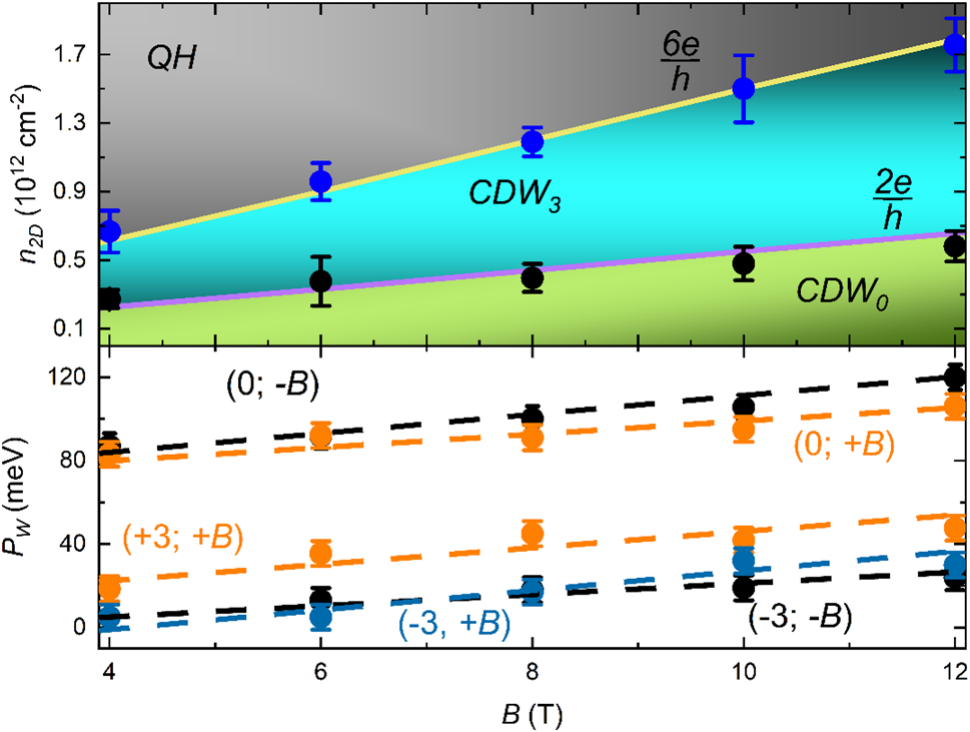
Magnetic field dependencies of plateau transition points and widths: top: Charge carrier densities $n_{2D}$ at which a transition from 0 $\to$ 3 $\frac{{e^{2} }}{h}$ to 3 $\to$ 6 $\frac{{e^{2} }}{h}$ are found (cf. Supplementary Note 4). Equivalent transition points at positive and negative conductivity and both magnetic field directions were averaged. The resulting standard deviation, the relevant error here, is reflected in the displayed error bars. Solid lines have the theoretical slope $\frac{2e}{h}$ and $\frac{6e}{h}$ corresponding to the increasing LLs degeneracy with *B*. Remarkably, the experimentally observed transition points from CDW_0_ to CDW_3_ and CDW_3_ to QH are in excellent agreement with both degeneracy lines. The indices denominate the transverse conductivity value, QH refers to the quantum Hall behavior. Bottom: Energy widths *P*_*w*_ of the plateaus $\sigma_{xy} = 0$ and $\pm 3\frac{{e^{2} }}{h}$ found for S3 plotted as a function of magnetic field strength (error bars account for the extended deflection at the plateau edges). The parentheses denote the respective $\sigma_{xy}$ value in units of $\frac{{e^{2} }}{h}$ and the relative magnetic field direction $\left( \pm \right)$. Dashed lines are guide to the eye.

The dependencies of the transition points between $\sigma_{xy}$ plateaus on the magnetic field strength reveal further insight into and consistencies within the observed CDW states which we term CDW_0_ and CDW_3_ for convenience (indices relate to the transverse conductivity value). Addressing here S3 with clearest developed features, we note that the 0 and $\pm 3\,\frac{{e^{2} }}{h}$ plateaus appear instantaneously for *B* $\ge 4\,T$ (cf. Supplementary Note 4). In particular, below this field strength no plateaus are observable (including quantum Hall plateaus). That is, the system directly energetically favors a CDW phase formation even in the limit of low $n_{2D}$. In Fig. 4 we address in more detail our observation of the plateau transitions 0 $\to$ 3 $\frac{{e^{2} }}{h}$ and 3 $\to$ 6 $\frac{{e^{2} }}{h}$. We extracted the respective $n_{2D}$ at the transitions and find that these fall perfectly onto the theoretically expected LL degeneracy evolution with increasing *B* field. That is, onto lines with slopes $\frac{2e}{h}$ and $\frac{6e}{h}$, respectively (top panel, Fig. 4). Such behavior is consistent with an orbitally quantized CDW state, where the CDW periodicity scales linearly with *B*^60^. Remarkably, the results show that the CDW states persists independent of the actual filling of a LL at all *B*. We furthermore extracted the energetic widths *P*_*w*_ of the $\sigma_{xy}$ = 0 and $3\,\frac{{e^{2} }}{h}$ plateaus (lower panel, Fig. 4). The observed widths exclude Zeeman splitting, as otherwise *B* > 700 T and > 170 T, respectively, would be necessary^44^.

## Discussion

The transitions between the CDW phases and to the quantum Hall phase (in the limit of filling of higher LLs) reflects the interplay of different screening sources and the magneto-induced localization in our system. Regarding screening, for small $n_{2D}$, only within the *n* = 0 LL, the potential modulation introduced by the TCNQ leads to a reduction of ϵ, thereby allowing the formation of a CDW. The screening experienced by the charge carriers within the *n* = 0 LL is therefore a combination of both the TCNQ-induced and the entirely filled hole-spectrum^59^. Upon further occupation of higher LLs, additional changes to the screening within the highest occupied LL from completely filled lower-lying LLs comes to play, too. This implies that the effective Coulomb interaction in every LL is principally differing. In particular our findings are consistent with lower-lying filled LLs tending to increase the screening for the higher ones^59^. In respect of the localization action of the magnetic field^40^, for all $n_{2D}$ below the transition to $\pm 3\frac{{e^{2} }}{h}$, $l_{B}$ is smaller or comparable in magnitude to $r_{s}$, that is, CDW formation is principally supported in this $n_{2D}$ range. In other words, the CDW_0_ is supported by both, the screening and the magneto-induced localization. Beyond the transition, in the CDW_3_ regime, $\frac{{l_{B} }}{{r_{S} }}$ is of the order of unity, that is, the magnetic localization is less pronounced. However, we still observe the CDW signature, suggesting that the electron–electron interactions are sufficiently strong to favor charge ordering despite the weaker localization. Only when $\frac{{l_{B} }}{{r_{S} }} \sim 2$, the transition to the quantum Hall phase with $\sigma_{xy} = \pm 6\frac{{e^{2} }}{h}$ and vanishing $\rho_{xx}$ is found. The interplay of screening and magnetic field localization thus explains the sequence of plateaus and the transition from CDW to the quantum Hall phase with the occupation of higher LLs. The magnetic length furthermore puts a physically reasonable lower limit of $2l_{B}$ for the CDW periodicity, which is also consistent with the circumstance that the CDW phases persist at any individual LL filling. For completeness, within the context of CDW formation, we note that the weakly developed plateau signature at $\sigma_{xy} = 0\frac{{e^{2} }}{h}$ in S1 provides the opportunity for an estimate of the critical impurity concentration $n_{i}^{\left( c \right)}$ in our system. Noting that the transition to the $\sigma_{xy} = \pm 6\frac{{e^{2} }}{h}$ quantum Hall state occurs already at comparably low $n_{2D}$, implies that the ionized TCNQ concentration of S1, $n_{i}^{{\left( {\mathrm{S1}} \right)}} = 1.67 \times 10^{10} \;{\text{cm}}^{ - 2}$ , is close to $n_{i}^{\left( c \right)}$. That is, one can estimate $n_{i}^{\left( c \right)} \gtrsim 2 \times 10^{10} \;{\text{cm}}^{ - 2}$ for our specific system. We further note, that the type of density wave formed can principally be influenced by the choice of adsorbent. For instance, if by choice, the adsorbent-lattice interaction is sufficiently strong, the 2D Weyl-like nature of fermions can be deliberately shifted to the full Dirac fermion picture. Therefore our study demonstrates a general pathway to generate and investigate theorized and unknown CDWs or other macroscopic quantum states in low-D Dirac and Weyl(-like) fermions, and access the transition and competition between such quantum phases.

## Methods

Graphene flakes were obtained through standard mechanical exfoliation and a subsequent heating step on a SiO_2_/Si substrate, followed by the deposition of TCNQ on top. TCNQ (98% purity, purchased from Merck KGaA) was evaporated from powder in vacuum (*p* $\approx 1 \times 10^{ - 4}$ mbar) in a thermal evaporation chamber. The system was preheated to 60 °C for 30 min, then the TCNQ was evaporated at 105 °C for different times to vary the amount of TCNQ. Standard micro-Raman spectroscopy under ambient conditions (laser wavelength $\lambda$ = 532 nm) was used to characterize the samples before and after TCNQ deposition (cf. Supplementary Note 2). The samples were then electrically contacted in standard Hall-bar geometry via electron-beam lithography using palladium as electrode material. Electrical measurements were performed in a standard cryostat system at liquid Helium temperatures equipped with a superconducting magnet. The used devices were Keithley 2400 power supplies to apply the bias and the gate voltage, a SR570 current preamplifier and Keithley 2000 digital multimeters to measure simultaneously the four-point voltage and the preamplifier output.

## Supplementary Information

Supplementary Information.

## Data Availability

The data that support the findings of this study are available from the corresponding author upon reasonable request.
