# Supplementary material for: Fingerprints of magnetoinduced charge density waves in monolayer graphene beyond half filling
Source: Sci Rep. 2022 Dec 15;12:21664. doi: 10.1038/s41598-022-26122-0 (PMC9755137; doi:10.1038/s41598-022-26122-0)
Supplement: Supplementary file 1 — Supplementary Information. [file 41598_2022_26122_MOESM1_ESM.pdf]

Supplementary Information for

Fingerprints of magnetoinduced charge density waves in monolayer graphene beyond half filling

Felix Hoffmann, Martin Siebert, Antonia Duft, and Vojislav Krstić*

Department of Physics, Friedrich-Alexander-University (FAU) Erlangen-Nürnberg, Staudtstraße 7, 91058 Erlangen, Germany

I. Supplementary Note 1

The formation of a CDW in 2D can be derived from an extended Hubbard Hamiltonian of the form

$$H = t \sum_{\langle ij \rangle, \alpha} \left[c_{i,\alpha}^{\dagger} c_{j,\alpha} + H.c. \right] + U \sum_{i} n_{i\uparrow} n_{i\downarrow} + V \sum_{\langle ij \rangle} n_i n_j$$

which is parametrized by the mutual Coulomb interaction V, the on-site energy U and the hopping integral t. Here $\langle ij \rangle$ stands for the lattice sites i and j, α denotes spin orientations and $n_i n_j$ is the number operator. The relative magnitude of the mutual Coulomb interaction V can be described by the ratio $\frac{E_C}{E_K}$ of the pairwise Coulomb repulsion energy E_C and the particles' kinetic energy E_K, which scales inherently with t. The existence of a CDW due to generic charge ordering can then be derived within this framework depending on the increase of the energy ratio $\frac{V}{U}$. In other words, once E_C prevails over E_K the development of a CDW becomes more favorable. This relation can also be derived by considering the timescale on which an interaction between two charge carriers can occur. In a very simple picture, the time two charge carriers are in close proximity to each other is related to the kinetic energy as $t_K \propto \frac{1}{E_K}$. Similarly, the time for a Coulomb interaction to take place is

inversely proportional to the Coulomb repulsion energy $t_C \propto \frac{1}{E_C}$ (as $E_C \cdot t_C \geq \frac{\hbar}{2}$). It is reasonable to assume, that a stronger interaction can occur, when

$$t_K \gtrsim t_C \qquad \text{and therefore} \qquad \frac{1}{E_K} \gtrsim \frac{1}{E_C}.$$

By rearrangement one finds

$$\frac{E_C}{E_K} \gtrsim 1.$$

Within this ratio lies furthermore an important difference between mass-carrying and massless charge carriers. The Coulomb repulsion is proportional to $\epsilon^{-1}\sqrt{n_{2D}}$, where n_{2D} is the charge carrier density and ϵ is the dielectric permittivity associated with the screening properties of the system. Since for mass-carrying charge carriers Since for mass-carrying charge carriers $E_K \propto n_{2D}$, one finds

$$\frac{E_C}{E_K} \propto \frac{1}{\epsilon\sqrt{n_{2D}}}.$$

Therefore, by simply adjusting n_{2D} through, for instance, electrostatic gating, such system can be tuned experimentally into a CDW-favorable condition [23]. In contrast, for massless fermionic charge carriers $E_K \propto \sqrt{n_{2D}}$. Consequently, the relative strength of the effective Coulomb interaction

$$\frac{E_C}{E_K} \propto \frac{1}{\epsilon} \qquad (2)$$

is independent of n_{2D} and solely depends on the screening properties of the system [S1].

II. Supplementary Note 2

All experimentally acquired Raman spectra were analyzed using individual fits with the Lorentz function to the relevant peaks. In order to calculate the TCNQ induced charge carrier density the G-band position was analyzed with respect to the theoretically expected G-band position for pristine graphene. This is necessary as already before TCNQ deposition a non-negligible doping is observable in the pristine samples due to a slightly different coupling to the substrate [S1]. The difference between the position before and after TCNQ deposition is then related to the molecule and can be used to estimate the Fermi energy shift and subsequently the induced charge carrier density using a conversion factor obtained from [S2].

III. Supplementary Note 3

The discussed CDW signature of a maximal longitudinal resistivity ρ_{xx} appearing together with unconventional plateaus in transverse conductivity σ_{xy} can in principal also be attributed to concurring phenomena such as the quantum Hall insulator (QHI) [S3,S4], the magnetic catalysis [S5-S8] or Zeeman splitting [S9-S11]. However, the unambiguous distinction between these phenomena and a CDW phase is straightforward in the specific case of the $\sigma_{xy} = 0\frac{e^2}{h}$ plateau. Regarding the QHI it suffices to consider the extent of the plateau. The QHI only exists and collapses within the lowest Landau level and is followed by the conventional sequence of quantum Hall plateaus (that is $\sigma_{xy} = \pm2\frac{e^2}{h}$, $\pm6\frac{e^2}{h}$ ... in graphene) with the respective minima in ρ_{xx} [S3,S4]. Similarly, the magnetic catalysis exclusively reveals plateaus in σ_{xy} showing dissipationless longitudinal transport (except at the charge neutrality point where $\sigma_{xy} = 0\frac{e^2}{h}$), that is, common quantum Hall states. Furthermore, the mechanism excludes, besides $\pm1\frac{e^2}{h}$, all other odd σ_{xy} plateau values [S6-

S9]. Finally, the Zeeman splitting can be tested through the energy width of the $0\frac{e^2}{h}$ plateau at a given magnetic field strength. The energetic splitting magnitude of Zeeman splitting is given by [S12]

$$E_Z = g^* \cdot \mu_B \cdot B$$

where μ_B is the Bohr magneton, B is the magnetic field and g^* is the Landé factor. Under the assumption that g^* in graphene is the same as in vacuum (g^* = 2) [S12] , the magnetic field strength necessary to achieve plateaus with energy widths of $P_W \approx 20\,\text{meV}$ or $P_W \approx 80\,\text{meV}$ can be calculated to be >170 T or >700 T, respectively.

IV. Supplementary Note 4

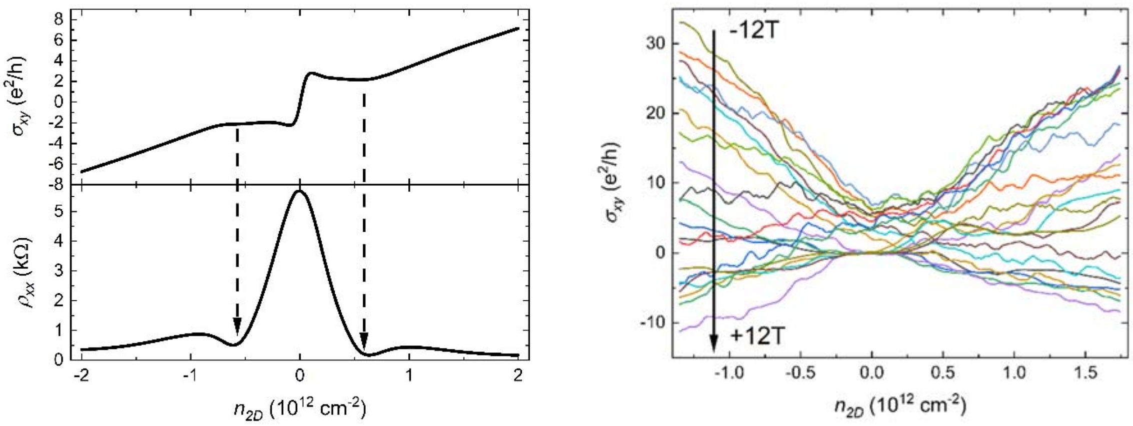

Figure 1. Electrical data for the reference sample and sample S3. (a) Magnetotransport measurement of the reference sample at 200 K. Distinct plateaus at $\pm 2\frac{e^2}{h}$ are found in the Hall conductivity σ_{xy} (top), accompanied by minima in the longitudinal resistivity ρ_{xx} (bottom). This fulfills the hallmarks for the quantum Hall effect in graphene [S13-S16]. (b) Measurements of the Hall conductivity σ_{xy} of S3 with respect to the charge carrier density

for magnetic fields ranging from -12 T to +12 T. The σ_{xy} $=0\frac{e^2}{h}$ plateau at the charge neutrality point is well pronounced for magnetic field strengths greater than 4 T.

We performed magnetotransport measurements at room temperature and a magnetic field strength of 12 T for the reference sample at 200K (without TCNQ) (cf. Fig. 1a). The sample clearly shows plateaus at $\pm\, 2\frac{e^2}{h}$ in the Hall conductivity σ_{xy}, accompanied by minima in the longitudinal resistivity ρ_{xx}. This presence of quantum Hall states [S13-S16] at moderate temperature demonstrates that our fabrication process is non-invasive and results in high quality graphene samples.

In Fig. 1b we show a summary of the Hall conductivities obtained from magnetotransport measurements performed on Sample S3 with magnetic fields strengths ranging from -12 T to 12 T. The zero conductivity plateau at the CNP as well as the unconventional plateaus at $\sigma_{xy} = \pm 3\frac{e^2}{h}$ are observable only for magnetic field strengths > 4 T.

V. Supplementary Note 5

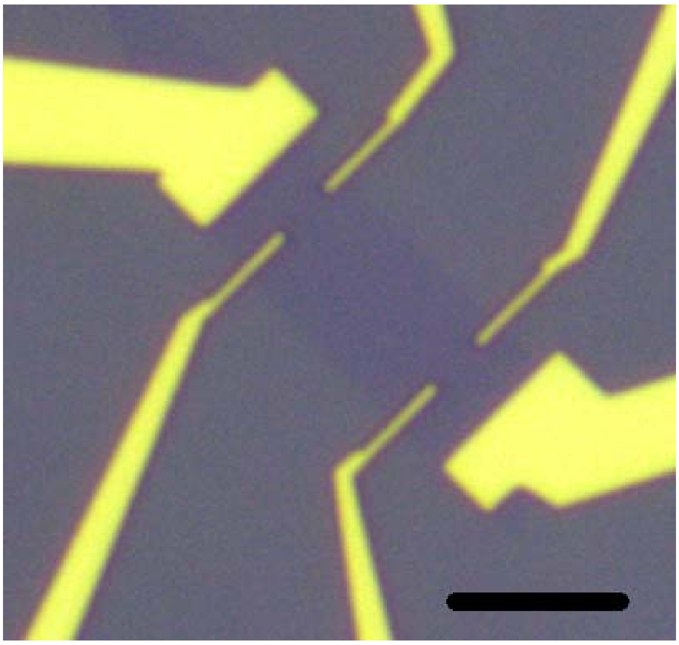

Figure 2. Optical image of an exemplary sample after device fabrication. The scale bar is 5 μm.

References:

[S1] Kang, Y.-J., Kang, J. and Chang, K.J. Electronic structure of graphene and doping effect on SiO2. *Phys. Rev. B* **78**, 115404 (2008).

[S2] Yan, J., Zhang, Y., Kim, P. & Pinczuk, A. Electric field effect tuning of electron-phonon coupling in graphene. *Phys. Rev. Lett.* **98**, 166802 (2007).

[S3] J. G. Checkelsky, L. Li, and N. P. Ong, Phys. Rev. Lett. 100, 206801 (2008).

[S4] J. G. Checkelsky, L. Li, and N. P. Ong, Phys. Rev. B 79, 115434 (2009).

[S5] X. Du, I. Skachko, F. Duerr, A. Luican, and E. Y. Andrei, Nature 462, 192 (2009).

[S6] K. Yang, Solid State Communications 143, 27 (2007).

[S7] Y. Zhao, P. Cadden-Zimansky, F. Ghahari, and P. Kim, Phys. Rev. Lett. 108, 106804 (2012).

[S8] S. Das Sarma and K. Yang, Solid State Communications 149, 1502 (2009).

[S9] Y. Zhang, Z. Jiang, J. P. Small, M. S. Purewal, Y.-W. Tan, M. Fazlollahi, J. D. Chudow, J. A. Jaszczak, H. L. Stormer, and P. Kim, Phys. Rev. Lett. 96, 136806 (2006).

[S10] D. A. Abanin, P. A. Lee, and L. S. Levitov, Phys. Rev. Lett. 96, 176803 (2006).

[S11] D. A. Abanin, K. S. Novoselov, U. Zeitler, P. A. Lee, A. K. Geim, and L. S. Levitov, Phys. Rev. Lett. 98, 196806 (2007).

[S12] S. Das Sarma, S. Adam, E. H. Hwang, and E. Rossi, *Rev. Mod. Phys.* 83, 407 (2011).

[S13] A. H. Castro Neto, F. Guinea, N. M. R. Peres, K. S. Novoselov, and A. K. Geim, Rev. Mod. Phys. 81, 109 (2009).

[S14] K. S. Novoselov, A. K. Geim, S. V. Morozov, D. Jiang, Y. Zhang, S. V. Dubonos, I. V. Grigorieva, and A. A. Firsov, Science 306, 666 (2004).
[S15] N. M. R. Peres, Rev. Mod. Phys. 82, 2673 (2010).
[S16] C. Rao, A. Sood, K. Subrahmanyam, and A. Govindaraj, Angew. Chem. Int. Ed. 48, 7752 (2009).
